# Circadian changes in the composition of human milk macronutrients depending on pregnancy duration: a cross-sectional study

**DOI:** 10.1186/s13006-020-00291-y

**Published:** 2020-05-25

**Authors:** Ieva Jura Paulaviciene, Arunas Liubsys, Alma Molyte, Audrone Eidukaite, Vytautas Usonis

**Affiliations:** 1 Clinic of Children Diseases, Institute of Clinical Medicine, Faculty of Medicine, Vilnius University, Vilnius, Lithuania; 2 Vilnius University Hospital Santaros Klinikos, Vilnius, Lithuania; 3 Department of Human and Medical Genetics, Institute of Biomedical Sciences, Faculty of Medicine, Vilnius University, Vilnius, Lithuania; 4 Department of Information Systems, Faculty of Fundamental Sciences, Vilnius Gediminas Technical University, Vilnius, Lithuania; 5 State Research Institute, Center of Innovative Medicine, Department of Immunology, Vilnius, Lithuania

**Keywords:** Circadian variation, Human milk, Macronutrient, Mid-infrared spectrophotometry

## Abstract

**Background:**

The purpose of this study was to evaluate the circadian variation of human milk macronutrients and energy content depending upon pregnancy duration.

**Methods:**

One hundred eighty fresh human milk samples from 45 mothers (27 of preterm and 18 of full-term newborns) were collected on a single day chosen between the 14th to 16th day after delivery. The samples were taken four times per day at 12 PM, 6 PM, 12 AM and 6 AM. Only lactating women, who could not breastfeed their hospitalized newborns and expressed milk by breast pump, were enrolled in the study. Human milk macronutrient composition and energy count were evaluated by mid-infrared spectrophotometry.

**Results:**

Significant differences in macronutrient content were observed between 6 AM and 12 PM for mean protein content (*t* = − 4.62, *df* = 44, *p* < 0.001), for mean fat content (*t* = − 2.10, *df* = 44, *p* = 0.04) and for mean energy content (*t* = − 2.24, *df* = 44, *p =* 0.03); between 6 AM and 6 PM for mean protein content (*t* = − 2.41, *df* = 43, *p* = 0.02), for mean fat content (*t* = − 3.76, *df* = 43, *p =* 0.001) and for mean energy content (*t* = − 3.85, *df* = 43, *p <* 0.001); between 12 PM and 12 AM for mean protein content (Wilcoxon test V = 75.5, *p* = 0.001), for mean fat content (*t* = 2.50, *df* = 44, *p* = 0.02) and for mean energy content (*t* = 2.74, *df* = 44, *p =* 0.01); between 6 PM and 12 AM for mean protein content (V = 229, *p* = 0.02), for mean fat content (*t* = 4.39, *df* = 43, *p <* 0.001) and for mean energy content (*t* = − 4.57, *df* = 43, *p <* 0.001). The average content of carbohydrates did not change significantly during the 24 h. The samples of preterm newborns’ mothers had more apparent diurnal fluctuations in macronutrient content.

**Conclusions:**

Our study revealed significant diurnal variations in protein and fat in human milk, and these circadian fluctuations were more apparent in the milk of mothers of preterm infants.

## Background

Human milk is a dynamic biological liquid whose composition varies in response to many factors, matching individual infant requirements. It is likely that changes in human milk composition are essential for health and growth, as well as for the development of infants. Each woman’s milk composition is unique. Factors that can influence human milk composition may be maternal (e.g., metabolic health, diet, and obstetric history), infant (e.g., gender, gestational age, and birth weight), physiological (e.g., stage of lactation, circadian rhythm, and stage of nursing), behavioural (e.g., time between feedings and manual or breast pump expression), and methodological (e.g., choice of analytical procedures, freeze–defrost cycles) [1–4].

Human milk macronutrient composition has been well analysed in correlation with increasing lactation time [2, 5–7], while less is known about its circadian changes. According to the available knowledge, diurnal changes mostly affect lipids [8–11], while data regarding other macronutrients remain conflicting. Although there are discrepancies in the composition of milk during different lactation periods between the mothers of preterm and term infants [2, 5], there is still a paucity of knowledge about the circadian differences in milk composition between these two groups. Additionally, it is not clear whether the circadian variation of human milk macronutrient content has potential benefits for infants’ health and well-being [12].

Since human milk composition strongly depends on the type of samples collected [1, 4, 13], the milk sampling procedure should be performed under standardised conditions, with the corresponding method chosen according to the purpose of the analysis. It has been shown that the mean concentration of total fat in milk obtained by an electric pump is higher than that of hand-expressed milk [4]. Electric pump use is recommended for the regular expression of milk, and the hand-operated pump could be the second choice. Hand expression is not recommended, and drip milk is not suitable for obtaining representative milk samples [14]. The macronutrient content varies depending on the degree of breast fullness; therefore, the breast should be entirely emptied, and milk should be mixed before analysis to obtain reliable results [1, 13, 15].

The purpose of our study was to test the circadian changes in human milk macronutrients and energy content among women who delivered their newborns at different gestational ages by mid-infrared spectrophotometry using a Miris Human Milk Analyser.

## Methods

The cross-sectional study was conducted from October 2017 to May 2018 at the Neonatal Center of Vilnius University Hospital Santaros Klinikos. It is one of two perinatal centres in Lithuania where the majority of very low and extremely low birth weight newborns are concentrated from all over the country. The study was approved by the local bioethics committee (permission No. 158200–17–925-443). Before being enrolled in the study, all participating women provided written informed consent.

Women were eligible to participate in the study if they were healthy (no history of diabetes, hepatitis B or C, HIV, tuberculosis, mastitis, or oncological diseases), had a single-birth pregnancy and were not on a special diet. Only lactating mothers who could not breastfeed their newborns due to the baby’s medical condition (either prematurity or disease) but who expressed milk that could be used for the feeding of their own babies were enrolled in the study. Breastfeeding mothers were excluded from the study for ethical reasons (for minimising nutritional or behavioural disruption to the mother and the newborn) and to avoid the influence of previous breastfeedings on the human milk macronutrient content during milk collection for analysis.

**Fig. 1:**
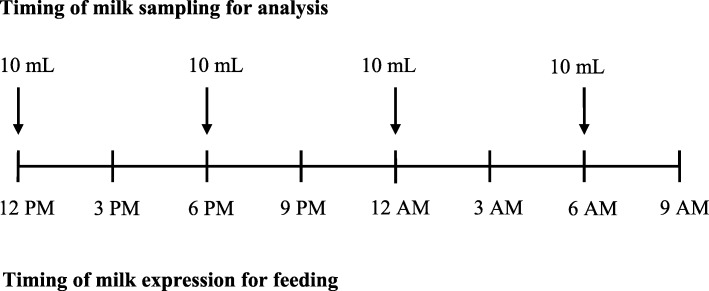
Timing of milk expression and sample collection

Verbal instructions for milk sample collection and appropriate use of milk pumps were given to the women at the beginning of the enrolment in the study. Milk samples from 45 mothers (27 of preterm and 18 of full-term newborns) were collected on a single day chosen between the 14th and 16th day after delivery. The samples were taken four times during the chosen day at the collection points of 12 PM, 6 PM 12 AM and 6 AM (±1 h). This lactation period was chosen for milk analysis due to the stability of maternal milk production and the ability of the mothers to have sufficient milk to donate some for analysis. Expressing milk every 3 h before feeding the babies is a common practice among mothers at our neonatal centre; therefore, the particular hours for milk sampling were chosen to not disrupt their daily milk expression routine. A timeline summarising the timing of milk expression for feeding versus samples for analysis over the course of the day is presented in Fig. 1.

After one or both breasts were fully emptied with an electrical or manual breast pump and the milk was carefully mixed in the container, samples of 10 mL milk were immediately collected in separate sterile plastic containers. The remaining amount of the expressed fresh milk was used to feed the newborns. After being labelled (ID number, data and correct time of collection), each sample was stored in a refrigerator at + 4 °C for further analysis of the milk’s macronutrient composition. The majority of the milk samples (44 out of 46 participating women) were analysed 22 to 25 h after the beginning of the collection (at 12 PM), while the samples of two mothers were collected during the weekend and stored in the refrigerator up to 48 h before being analysed (due to the day off of the milk bank).

The milk’s macronutrient and energy content was evaluated by mid-infrared spectrophotometry using the Miris Human Milk Analyser (Miris AB, Sweden), which was operated using the calibration mode for processed (homogenised) milk. According to the manufacturer’s instructions, prior to analysis, the milk samples were warmed to 40 °C and homogenised for 1.5 s/mL using the Miris Ultrasonic Processor. A daily calibration check was performed using the calibration solution (MIRIS check), which was provided by the supplier.

Statistical analysis was performed using the R program version 3.4.4. Data were summarized as the mean ± standard deviation. The Chi-square and exact Fisher test were used to establish significant differences between the groups. The Shapiro-Francia test was used to determine the normality of the data. In order to compare the two groups, we used the Student t test when the data were normally distributed. In other cases, we used the Wilcoxon test to compare non-parametric groups. Cohen’s d effect size for t test was calculated. Cohen’s d values between ≥0.2 and < 0.5 indicate small-size effect, values between ≥0.5 and < 0.8 indicate medium-size effect, and values ≥0.8 indicate large-size effect [16]. Statistical significance was achieved when the *p* value was < 0.05.

## Results

A total of 180 samples from 45 lactating mothers were collected and analysed for macronutrient and energy content. All participating women were permanent residents of Lithuania. The main characteristics of the women enrolled in the study are presented in Table 1. The mothers of term and preterm newborns were similar regarding newborn gender, delivery mode, age, ethnicity and number of deliveries.

**Table 1:** Characteristics of the mothers of preterm and term newborns

Characteristics	Term (GA ≥37 weeks), *n* = 18	Preterm (GA < 37 weeks), *n* = 27	*t*	*df*	*p* value
Gestational age (weeks), $\overline{x}\pm s$ Range (weeks)	38.7 ± 1.0 37–40	30.2 ± 2.5 24–36	−15.61	37.04	< 0.001
Birth weight (g), $\overline{x}\pm s$ Range (g)	3265 ± 589 1590–3960	1477 ± 405 845–2380	−12.09	43	< 0.001
Gender (male / female), n (%)	11/7 (61.1/38.9)	18/9 (66.7/33.3)	0.15	1	0.703
Delivery mode (VD / CS), n (%)	15/3 (83.3/16.7)	16/11 (59.3/40.7)	2.92	1	0.087
Number of deliveries, $\overline{x}\pm s$ Range, n	1.6 ± 0.7 1–3	1.7 ± 0.9 1–5	0.22	43	0.829
Maternal age (y), $\overline{x}\pm s$ Range (y)	30.9 ± 6.2 19–40	32.9 ± 4.3 24–43	1.28	43	0.209
Ethnicity (Lithuanian, other^a^), n (%)	14/4 (77.8/22.2)	23/4 (85.2/14.8)	0.25	1	0.694

$\overline{x}$ ± *s* mean value ± standard deviation, *VD* vaginal delivery, *CS* caesarean section, *GA* gestational age; *t* test value, *df* degrees of freedom

^a^other ethnicities: Russians, Poles, Ukrainians

Analysis of all 180 samples from the 45 women enrolled in the study showed significant diurnal variation of protein, fat, and energy content, with the highest levels of these macronutrients and energy content during day expressions (at 12 PM and 6 PM) and with the lowest during night expressions (at 12 AM and 6 AM). The carbohydrate content in the human milk did not reveal significant diurnal fluctuations (Table 2).

**Table 2:** Macronutrient and energy content in preterm and term human milk (*n* = 45) during the daytime

		Time of day (hours)					
		6 AM – 12 PM	6 AM – 6 PM	6 AM – 12 AM	12 PM – 6 PM	12 PM – 12 AM	6 PM – 12 AM
Protein, g/100 mL	$\overline{x}\pm s$	1.37 ± 0.2 1.43 ± 0.2	1.37 ± 0.2 1.41 ± 0.2	1.37 ± 0.2 1.37 ± 0.2	1.43 ± 0.2 1.41 ± 0.2	1.43 ± 0.2 1.37 ± 0.2	1.41 ± 0.2 1.37 ± 0.2
	*p* value	< 0.001^**^	0.02^*^	0.90	0.24	0.001^*^	0.02^*^
	Cohen’s d	0.30^a^	0.20^a^	0	0.10	0.30^a^	0.20^a^
Fat, g/100 mL	$\overline{x}\pm s$	4.06 ± 0.9 4.39 ± 1.1	4.06 ± 0.9 4.57 ± 1	4.06 ± 0.9 4.00 ± 0.7	4.39 ± 1.1 4.57 ± 1.0	4.39 ± 1.1 4.00 ± 0.7	4.57 ± 1.0 4.00 ± 0.7
	*p* value	0.04^*^	0.001^*^	0.65	0.28	0.02^*^	< 0.001^**^
	Cohen’s d	0.33^a^	0.50^b^	0.07	0.17	0.42^a^	0.66^b^
Carbohydrate, g/100 mL	$\overline{x}\pm s$	7.39 ± 0.2 7.33 ± 0.3	7.39 ± 0.2 7.33 ± 0.2	7.39 ± 0.2 7.37 ± 0.2	7.33 ± 0.3 7.33 ± 0.2	7.33 ± 0.3 7.37 ± 0.2	7.33 ± 0.2 7.37 ± 0.2
	*p* value	0.12	0.14	0.40	0.88	0.27	0.22
	Cohen’s d	0.24^a^	0.24^a^	0.08	0	0.16	0.20^a^
Energy, kcal/ 100 mL	$\overline{x}\pm s$	74.42 ± 9.1 77.58 ± 10.0	74.42 ± 9.1 79.18 ± 9.7	74.42 ± 9.1 73.89 ± 6.8	77.58 ± 10.0 79.18 ± 9.7	77.58 ± 10.0 73.89 ± 6.8	79.18 ± 9.7 73.89 ± 6.8
	*p* value	0.03^*^	< 0.001^**^	0.59	0.29	0.01^*^	< 0.001^**^
	Cohen’s d	0.33^a^	0.51^b^	0.07	0.16	0.43^a^	0.63^b^

$\overline{x}$ ± *s* mean value ± standard deviation; Cohen’s d – Cohen’s d effect size

^a^ – small effect size, ^b^ medium effect size; significant differences are highlighted in bold (^*^p < 0.05; ^**^p < 0.001)

**Fig. 2:**
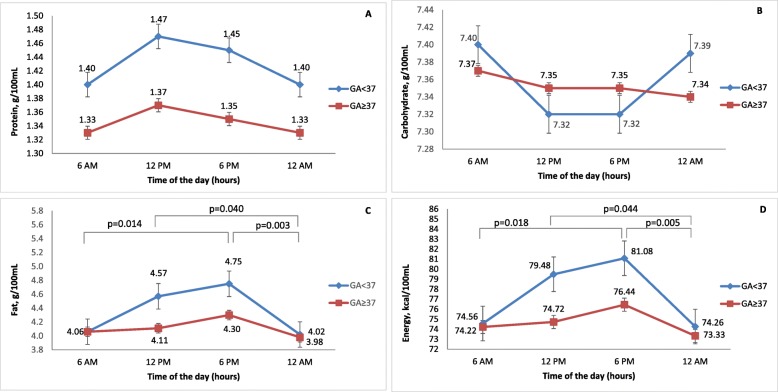
Comparison of macronutrient content in preterm (*n* = 18) and term (*n* = 27) human milk by time of day; GA – gestational age (weeks). Significant differences in fat and energy content were found only in the preterm group (GA < 37)

A comparison of macronutrient content changes in preterm and term human milk separately during the time of the day is presented in Fig. 2. Macronutrients and energy content did not differ significantly when comparing the preterm and term human milk samples, but the diurnal variations were more pronounced in the preterm milk samples.

Although protein content revealed similar diurnal fluctuations in both the preterm and full-term milk samples, with the highest levels during day expressions (at 12 PM and 6 PM), these differences were not significant. The preterm milk samples also contained more protein than the full-term samples, but the differences were not significant (Fig. 2a).

Carbohydrate content did not show apparent diurnal fluctuations in the full-term samples but showed more apparent diurnal fluctuations in the preterm milk samples, although these differences did not reach significance. In contrast to the case for proteins, the highest concentration of carbohydrates in preterm milk was observed during night expression, and the lowest was observed during the day (Fig. 2b).

We found significant differences in the diurnal fluctuations of fat and energy, with peak concentrations at 12 PM and 6 PM in the preterm milk samples, but these differences did not reach significance in the full-term milk samples (Fig. 2c and d).

## Discussion

Our study is the first to investigate circadian changes in human milk macronutrients in Lithuania. Circadian changes in human milk composition have already been analysed in a number of studies, but the presented results are very heterogeneous due to the different designs, number of subjects, and methods used for milk analysis. The previous studies found in PubMed are summarised in Table 3.

**Table 3:** Summary of the studies on circadian changes in human milk macronutrient content

Reference	Site	Participants (number)	Lactation stage	Macronutrients investigated	Analytical methods	Results
Gunther M et al., 1949 [8]	Germany	8	8–11 days of lactation	Fat	Gerber	The highest concentrations in the morning and at noon and the lowest between 8 PM and 4 AM
Prentice A et al., 1981 [17]	Gambia	60	1–18 months after delivery	Fat	CMT	A marked diurnal variation (the highest values in the early morning, the lowest in the late afternoon)
Harzer G et al., 1983 [18]	Germany	17 (13 German and 4 English)	First 5 weeks of lactation	Lipid	Thin-layer chromatography	Milk samples from German mothers had their peak lipid content in the afternoon (noon to 6 PM), while the English samples had their respective peaks in the evening (6 PM to midnight)
Lavine ME et al., 1986 [19]	US	6 (mothers of term infants)	8th week of lactation	Nitrogen, lipid	Micro-Kjeldahl method (nitrogen), modified Folch procedure (lipid)	Total nitrogen remained fairly constant during the day; total lipid exhibited significant circadian variation, with peak concentration in the morning (10.00 h) and afternoon (14.00 h) samples
Clark RM, 1987 [20]	US	7 (mothers of term infants)	8th week of lactation	Protein, urea nitrogen, free amino acids (taurine, glutamine, glutamic acid)	Micro-Kjeldahl method (total nitrogen) Crocker method (urea nitrogen)	Concentrations of nitrogen substances in the milk differed significantly among women but were relatively constant during the day
Jackson DA et al., 1988 [21]	Thailand	25 (mothers of term infants)	From the 1st to 9th months after delivery	Fat	CMT	Significant circadian variation, with maximum values between 16.00–20.00 h and minimum values between 04.00–08.00 h
Lammi-Keefe CJ et al., 1990 [22]	US	6 (mothers of term infants)	8 weeks after delivery	Lipid, carbohydrate, protein, calories	Modified Folch procedure (lipid), YSI model 27 Industrial Analyser (lipid), micro- Kjeldahl method (protein), Southgate-Durnin equation (calories)	Significant circadian variation of protein content, with maximum values at 6 AM and 6 PM. None of the other components varied significantly.
Stafford J, 1994 [23]	Mexico	10	Not specified	Lipid	Modified Folch method	Significant circadian variations of volume and lipid yield were noted, peaking at 8.00–12.00 and 16.00–20.00 h
Weber A et al., 2001 [24]	Germany	20 (mothers of VLBW infants)	The first 4 weeks of lactation	Protein, fat	Bicinchoninic acid method (protein), CMT (fat)	Fat but not protein was lower in morning samples than in samples collected later in the day
Lubetzky R et al., 2006 [10]	Israel	39 (mothers of preterm infants, 26–33 weeks)	7–14 days after delivery	Fat	CMT	CMT was significantly higher in evening (between 21.00 h and 24.00) than in morning (between 6.00 h and 9.00 h) samples
Lubetzky R et al., 2007 [9]	Israel	22 (mothers of preterm infants, 26–31 weeks)	2–7 weeks after delivery	Fat	CMT	Mean CMT was significantly higher in evening (9 PM to midnight) than morning samples (6–9 AM) during the first 7 weeks of lactation
Sanchez Lopez CL et al., 2011 [25]	Spain	69 (11 colostral group, 27 transitional group, 31 mature group)	< 2 months of lactation	Total nitrogen and protein content	Kjeldahl method	In the group of mature lactating women, protein content was significantly higher during the night-time (20.00 h – 8.00 h) than during the daytime (8.00–20.00 h)
Khan S et al., 2013 [26]	Australia	15 (mothers of term infants)	From the 1st to 6th months after delivery	Fat, lactose, total protein, casein, whey protein content	CMT (fat), enzymatic spectrophoto-metric method (lactose), Bradford protein assay (protein)	Fat content significantly differed over 24 h (higher during the day and lower at night, with no difference between morning and evening); the concentration of lactose and protein remained the same
Moran-Lev H et al., 2015 [11]	Israel	32 (mothers of preterm infants, 26–33 weeks)	2–7 weeks after delivery	Fat, carbohydrate, protein, energy	Mid-infrared transmission spectroscopy	Fat and energy contents during the whole period were significantly higher in evening samples; no significant differences between morning and evening in carbohydrates and protein contents
Çetinkaya AK et al., 2017 [27]	Turkey	52 (mothers of 30 preterm and 22 term infants)	5–15 lactation days (*n* = 27) and > 15 lactation days (*n* = 25)	Protein, fat, carbohydrate	Mid-infrared transmission spectroscopy	No significant difference was found in the protein, fat, and carbohydrate content of milk samples throughout the day
Hollanders JJ et al., 2019 [28]	Netherlands	10 (mothers of term infants)	1 month after delivery (± 5 days)	Cortisol, cortisone levels and fat, carbohydrate and protein content	Mid-infrared transmission spectroscopy	While in all the mothers, a diurnal rhythm of cortisol and cortisone could be seen, no rhythm appeared to be present for fat, carbohydrates, and protein

*US* United States, *VLBW* very low birthweight, *CMT* creamatocrit

There is yet no clear explanation but only suggestions and speculations of why these diurnal fluctuations of human milk composition exist and what impact they have on a newborn’s health and development. Hormonal changes in lactating women, breastfeeding patterns, the influence of the degree of breast fullness, circadian dietary habits, ethnic differences, and different techniques for the measurement of milk macronutrients are among the factors that can influence circadian changes in milk composition [9, 27, 29, 30]. However, the particular role of each factor and why and how these factors affect dynamic changes in specific macronutrients are unknown.

A number of methods for human milk macronutrient analysis have been used over the decades. Mid-infrared spectroscopy was the method of choice for human milk composition analysis in our study, as well as in a number of other studies [11, 27, 28]. There are some discrepancies, but there is also reliability comparing values of human milk macronutrients using mid-infrared spectroscopy and biochemical methods [31–34]. The main task of our study was to evaluate the dynamic changes in but not the absolute values of the macronutrient content of the human milk of mothers of preterm and full-term newborns during a 24-h period.

While most of the studies (as well as our study) show that the greatest circadian variation is in the fat content in human milk [9–11, 17, 21, 24], the data regarding other macronutrients remain controversial.

The DARLING study [29] showed that the macronutrient content in human milk could be influenced by breast fullness; human milk protein and fat concentrations were negatively related to milk volume, while milk lactose concentration was positively related to milk volume at certain lactation periods. In our study, the majority of the women usually rested at night and did not empty their breasts early in the morning (3 AM), so their breasts were full for the morning sampling (6 AM) compared to the midnight sampling. In spite of this, we did not find any differences in fat and protein content in the midnight and morning samples, different from other researchers.

The results regarding circadian fluctuations in protein content are still conflicting. Sánchez López and colleagues [25] found circadian changes in protein content during the mature milk stage with the highest protein concentrations during night (8 PM–8 AM) expression. In contrast to the Spanish study, we found the highest levels of protein during day expression (at 12 PM and 6 PM) and the lowest levels during night expression (at 6 AM and 12 AM). Keeping in mind that diurnal variations of the fat content of expressed milk had the same trend, we could only relate these fluctuations to the dietary habits of hospitalised women, i.e., day samples (at 12 PM and 6 PM) of milk were taken after breakfast and lunch meals, while night samples (at 12 AM and 6 AM) were taken on an empty stomach – relative to after a fasting period. Other investigators did not find any circadian changes in protein or nitrogen substance content, but the number of women participating in the studies was relatively small [11, 19, 20, 24, 26]. When analysing the preterm and full-term milk samples separately, we did not find significant differences in the circadian fluctuations of protein content in the two groups, although the preterm milk samples tended to show more apparent circadian variations of protein concentration than the full-term samples. We anticipate that the differences did not reach significance because of the small sample size of the preterm milk samples, despite the clear tendency of circadian variations.

Human milk carbohydrates showed the least variation in macronutrient concentrations over 24 h. Our results agree with those of previous studies showing no circadian variation in carbohydrate concentrations [11, 26, 27]. On the other hand, in our study, the preterm milk samples also tended to show more apparent circadian variations in carbohydrate content than the full-term samples.

We, like many other investigators, cannot find a reasonable explanation for the circadian changes of macronutrients in human milk but can only speculate with regard to the possible influence of the ethnicity or dietary habits of the mothers. It also seems that prematurity is another factor that may influence circadian fluctuations in human milk macronutrient content. It remains unclear, however, how these diurnal variations of human milk composition affect a newborn baby’s growth pattern and own biorhythms.

### Strengths and limitations

There are some limitations of our study.

Some of the women enrolled into the study could have preeclampsia and/or hypertension. Existing research shows that maternal preeclampsia or hypertension alters human milk composition [35–38], although there are very limited data on the influence of preeclampsia on human milk macronutrient content. Possible changes in human milk composition due to preeclampsia or hypertension may be a limitation of the generalizability of the study findings.

Analysis of the human milk macronutrient components was performed only during a short lactation period (i.e., 14–16 days after delivery), representing another limitation of our study. There are data showing that the circadian variation in human milk composition has different patterns throughout the evolution of the whole breastfeeding period [25, 39]. Therefore, the results of our study can only be applied to the transitional phase of the lactation period and cannot be adjusted for other phases of lactation. On the other hand, the analysis of the circadian variations of the macronutrient content of human milk of the mothers of preterm and full-term newborns at the same time after delivery could be the strength of our study. To the best of our knowledge, no other study has tried to find diurnal differences in human milk macronutrient composition after preterm and term childbirths during the same lactation period.

We analyzed human milk samples for macronutrient content four times a day and divided the participants into two subgroups. Many statistical tests were undertaken. In addition to *p* values, the effect sizes were calculated. The effect size should also be considered alongside estimation of the statistical significance: if the differences are significant (i.e., *p* < 0.05), but the effect size is small, the differences between the groups may be considered unimportant. A further limitation of our study is the deployment of multiple comparisons, without correction for this. We also acknowledge that our findings may not be generalizable to other populations.

To avoid the influence of different associated factors on the content of macronutrients in expressed human milk, we tried to unify the sampling process as much as possible. We accepted only samples obtained after full expression of one or both breasts using the breast pump and excluded breastfeeding mothers. Analysis of fresh but not frozen human milk samples using a Miris analyser made our material homogenous and reliable for comparison.

## Conclusion

Our study showed the circadian variability of human milk macronutrients, with the highest content of protein and fat during the day expressions (12 PM and 6 PM) and the lowest content during the night expressions (12 AM and 6 AM). There were no significant fluctuations in carbohydrate content in the human milk during 24 h. Moreover, the circadian fluctuations of the macronutrient content in human milk were more prominent following premature childbirth. Further research is needed to clarify the circadian changes in maternal milk during the whole lactation period and to determine whether preterm babies could benefit from these changes in human milk composition.

## Data Availability

The datasets used and analyzed during the current study are available from the corresponding author on reasonable request.
